# Culture, community, and cancer: Understandings of breast cancer from a non-lived experience among women living in Soweto

**DOI:** 10.21203/rs.3.rs-4797158/v1

**Published:** 2024-08-28

**Authors:** Seemela D. Malope, Shane A. Norris, Maureen Joffe

**Affiliations:** University of the Witwatersrand; University of Southampton; University of the Witwatersrand

**Keywords:** Breast cancer, Community perceptions Sociocultural influences, Health systems, Early detection, South Africa

## Abstract

**Background:**

Individual perceptions compounded with socio-cultural beliefs and health system factors are key determinants of people’s health seeking behavior and are widely cited as the causes of delayed breast cancer diagnosis among women from structurally vulnerable settings. Asking: “how do women with a non-lived experience of cancer understand the disease and, what informs their health seeking behaviors?”, we explored individual, sociocultural and health system elements from a conceptual model derived from the Socioecological, Health Belief and Cancer Stigma Frameworks, to understand perspectives of breast cancer in a South African urban community setting.

**Methods:**

Using a deductive approach, we conducted a qualitative study consisting of 6 focus group discussions among 34 women from Soweto, Johannesburg (aged 35–74 years) and followed-up with 20 semi-structured in-depth interviews.

**Results:**

Findings revealed some awareness of breast and other cancers, but confusion and gaps in understanding of the disease, resulting in socio-culturally influenced misperceptions of risks, causes, and outcomes following treatment of breast cancer. This fueled perceptions of profound fear and stigma against people with breast and other cancers. These findings together with participant perceptions of primary healthcare providers being unwelcoming, under-resourced, and insufficiently trained to deal with breast cancer, resulted in women reporting being reluctant to participating in screening/early detection care seeking behavior. They only accessed primary care when experiencing extreme pain or ill-health. Participants suggested as solutions for future interventions, the need for sustained community engagement, harnessing existing clinic and community stakeholders and resources to provide clear and understandable breast cancer information and encouragement for screening uptake.

**Conclusions:**

Health literacy gaps surrounding breast cancer fuels socio-culturally influenced misperceptions, fear, stigma, and fatalism among community women from Soweto, South Africa. Women perceive primary care providers of having insufficient knowledge, skills, and resources to provide effective breast cancer screening services. Participants suggested the need for greater community engagement involving primary clinics and existing community stakeholders working hand in hand. Clear, understandable, and consistent information about breast cancer must be regularly disseminated and communities must be regularly encouraged to utilise breast cancer screening services.

## Introduction

The global increase in cancer incidence and mortality has signaled a cancer pandemic. Cancers are now responsible for one in five deaths and rate second only to cardiovascular diseases in the global number of deaths by cause[1]. Though currently lower than in High-Income-Countries (HICs), cancer incidence is rapidly increasing in Low- and Middle-Income-Countries (LMICs)[1]. As of 2020, with an estimate of 2.3 million new cases and 684,996 global deaths, breast cancer is now the highest incident cancer in women and accounts for close to a quarter of all female cancers[2]. Today, a woman’s chances of being diagnosed with breast cancer is 1 in 8, an increase from the 1 in 11 almost 5 decades ago. While there have been great advances made in cancer therapy over the years, breast cancer trends and patterns including survival rates, vary from region to region due to the differences in exposure to risk factors. Survival patterns are also influenced by availability, accessibility, and affordability of screening, diagnostic and treatment cancer care services[3].

High-income countries (HICs) have a higher incidence of breast cancer, but low- and middle-income countries (LMICs) have relatively higher mortality rates for the disease[2, 4], such that their mortality to incidence ratios are double those of HICs. This is illustrated by the fact that whilst in 2020, some 8.3% of new cases occurred in Africa, the continents share of breast cancer deaths was much higher, i.e., 12.5% of the global deaths[2]. Currently more than 70% of breast cancer patients in HIC are diagnosed with early-stage disease (stages 1 and 2) because women in HIC have access to population-based mammography screening coupled with excellent diagnostic and treatment services which maximizes their 5-year survival rates to around 90%[5]. In contrast, an average two-thirds of breast cancer patients in LMICs are diagnosed with late-stage (3 or 4) disease, have generally sub-optimal treatment access and survival rates are lower[6]. The late-stage diagnosis in LMICs can be attributed to various factors such as poor knowledge and awareness of breast cancer including the risks of getting the disease, delays in seeking medical care, lack of cancer screening services and inadequate diagnostic and treatment resources[5–20].

Several studies[21–24] have called for more research to explore cancer from a sociocultural perspective because it considers how the disease is experienced from a social, cultural, and economical context. This is especially important in South Africa because social, cultural, and economical factors influence the way in which people respond to cancer. There have been a few studies conducted in South Africa over cancer perceptions. Mosavel and colleagues[23] conducted a study in Cape Town, South Africa wherein they explored narratives of mothers and daughters about the word “cancer”. The long-term aim of their research was to develop a cervical cancer intervention targeting both mothers and daughters. The findings for this study illustrated that cancer is greatly associated with death and suffering. The study further reported cancer fatalism, the belief that death is inevitable when a person has been diagnosed with cancer, along with fear and worry, also impact how mothers and daughters perceive cancer. In addition, cancer was thought of as having detrimental consequences by both mothers and daughters.

Zwane[25] conducted a culture-centered study examining breast cancer perceptions of breast cancer patients from underprivileged areas in Kwa Zulu Natal, South Africa. In this study, Zwane argues that “culture informs how people navigate every aspect of their life”. Findings from this study illustrated varying perceptions by isiZulu breast cancer patients with older women (45years old and above) having a more pragmatic response to their diagnosis and younger women having a more pessimistic response. Overall, patients reported the disease as physically and psychologically challenging; a shame inducing and expensive disease. In addition, participants reported a health system failure which contributed to a delay in treatment which increased the likelihood of the cancer metastasizing. Zwane further described the hospital as a structure that inhibits self-seeking action by setting up communication barriers. Bosire and colleagues[26] conducted a study in Soweto, South Africa which explored breast cancer survivors’ narratives of navigating comorbidity. Findings from this study highlighted discrimination and isolation explained through women’s fear of rejection by their loved ones as well as how their illnesses created social distance between their loved ones and the wider community.

What remains less documented is how breast cancer is understood and experienced from a non-lived experience by Black women who are at risk of developing the disease as this provides insight into background cultural and contextual factors around cancer, and breast cancer in particular, that may be significant influencing aspects for better detection and successful management of breast cancer. These perceptions of cancer and breast cancer may highlight the missing voices of women with a non-lived experience of cancer who are most likely to be heavily affected by the burden of the disease as they age. Therefore, the aim of the paper was two-fold: (i) Explore among women in Soweto, South Africa, their knowledge and individual, sociocultural and health system perceptions around cancer, and in particular breast cancer, and how they influence health service access for screening and early detection of breast cancer; and (ii) Elucidate opportunities and solutions to achieving earlier breast cancer detection and diagnosis in Soweto, South Africa.

### Conceptual Framework

The question posed in this study is: How do women with a non-lived experience of cancer understand the disease and, what informs their health seeking behaviors? We used an adaptation of the Socioecological model, the Health Belief Model (HBM) and the Cancer Stigma Framework[27–30] to explore cancer notions, nuances as well as, the social implications of breast cancer from an individual, community, sociocultural and the health system aspect as perceived and understood by women in Soweto. As one of the most widely applied theories of health behavior, the Health Belief Model posits that six constructs predict health behavior: risk susceptibility, risk severity, benefits to action, barriers to action, self-efficacy, and cues to action. The socioecological model is used to understand the dynamic interrelations among various personal and environmental factors. The HBM has been used in many studies with a wide range of cultural influences and health delivery systems to explain people’s perceptions of strategies or screening tests for cancer. The HBM suggests that a person’s belief in a personal threat of an illness or disease together with a person’s belief in the effectiveness of the recommended health behavior or action will predict the likelihood the person will adopt the behavior. It is more descriptive than explanatory and does not suggest a strategy for changing health-related action. The Cancer Stigma Framework examines stigma from an individual (internal), societal and health system perspective.

## Methods

### Setting

We conducted this study at Jabulani Safe Hub, a community center located in Jabulani, in Soweto, South Africa. All research participants were residents of Soweto, South Africa. With an estimated population of approximately 1.8 million, Soweto is arguably the largest black urban township in South Africa representing various ethnic identities, including Zulu, Sotho, Tswana, Tsonga, and others. We use the term “Black” to describe the study participants while acknowledging a problematic history of this identity as a political category instituted by apartheid to distinguish “Black” from “Coloured” and “White”[31]. Soweto is diverse economically, with middle-class neighbourhoods, working class communities, and informal settlements. Historically, Soweto has been documented as a major political and organizing center that has contributed significantly to research studies since the 1990s[32].

### Sampling

Six focus group discussions (FGDs) with an average of 5 women per group, 34 women Soweto residents aged 35–74 years of age, who had never been diagnosed with cancer provided written consented to participate in the FGDs, 20 of whom also participated in the subsequent in-depth interviews (IDIs). The participant selection and recruitment were carried out by SDM. Women from around several areas in Soweto (Mofolo, Rockville, Jabulani, Molapo) who believed they could provide insight on the various notions and perspectives on breast cancer were deemed eligible and were approached in-person and invited to participate. Further criteria for eligibility were that women could speak either Sesotho, Sepedi, isiZulu, or English. Posters describing the study and eligible participants along with contact numbers were also posted in several hair salons, local supermarkets, as well as community advertisement walls.

FGDs and in-depth interviews were conducted by the first author. An observer who also served as a note-taker was present during all discussions and interviews. FGDs were conducted over a period of 8 weeks between May and July 2022 with discussions and debriefs between each FGD; full saturation was reached by FGD 5 and was verified by FGD 6. In-depth interviews were subsequently conducted over a period of 2 weeks in April of 2023. Transcriptions and translations of all interviews were done by the first author.

**Fig 1:**
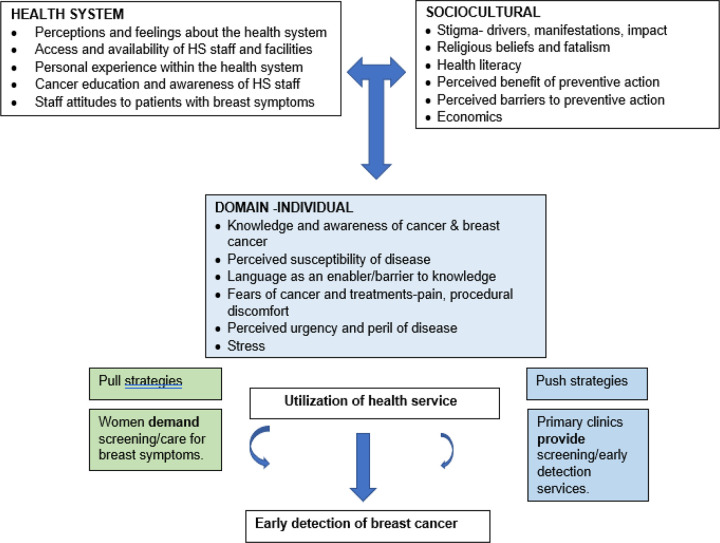
Conceptual framework using the socioecological model^1,2^ around the perceptions of cancer and specifically breast cancer with adaptations from the health belief model for why people utilize health services^2^ and cancer stigma conceptual framework^3,4^

FGDs were guided by the derived conceptual model (Fig. 1.) We explored how cancer is perceived understood, and experienced individually, socially, culturally, and within the primary healthcare services by women from Soweto, Johannesburg, South Africa.

The FGD approach was deemed to be appropriate for this study because it is a qualitative data collection method which entails a moderated interaction among a group of individuals to discuss individual experiences, beliefs, attitudes as well as perceptions with reference to a particular topic of interest[33]. FGDs are used to elicit information, meaning, and understanding of collective views and experiences[34]. FGDs enable qualitative researchers to explicitly explore the relationship between people’s perceptions and their socio-cultural situations; this is important because most people’s notions, understanding and interpretations are developed from experiential knowledge which comes from their immediate surroundings[33]. In-depth one-on-one interviews (IDIs) were subsequently conducted with some members of the FGDs to elicit their suggestions and solutions for early breast cancer detection provision (supply) by the health system and active utilization (demand) by community women. Both FGDs and IDIs were conducted using structured questionnaire guides (provided as supplementary tables 1 and 2). Questions asked were open-ended, allowing participants to answer fully and freely without any limits. Some of the responses given during the interviews probed follow-up questions which varied across all FGDs and in-depth interviews. Overall, while maintaining flexibility beyond pre-determined follow-up questions, the study was conducted to elicit rich and detailed accounts of specific topics of interests.

### Ethical approval

for this study was obtained from the University of the Witwatersrand, Johannesburg, South Africa, Human Research Ethics Committee (Medical), (M211023). Participant Information Sheets, Consent forms and Demographic sheets were provided and explained to participants before they agreed and provided written consent to participate in the discussions and agreed to the use of non-identifying quotes and to recording, transcription and translation of discussions. Pseudonyms were used throughout data transcription, translation and reporting to protect individuals’ confidentiality and ensure anonymity to the extent possible. Each participant was compensated with R300.00 (± USD 17) per interview for their time and to reimburse any transportation costs which might have been incurred. Refreshments were provided for FGDs and the in-depth interviews. The duration of each FGD ranged from 2 hours 20 minutes to 2 hours and 45 minutes. Subsequently, 20 of the 34 participants were randomly selected and invited back for follow-up interviews of 16 to 33 minutes duration.

### Data Analysis

The deductive analysis began with immersion of all FGD transcribed and translated recordings. Findings were then overlaid on the domains and elements of the conceptual model. Transcripts, fieldnotes and other textual documents were reviewed multiple times by the authors. Common and unique findings with supportive quotations from participants were identified and tabulated. Individual review and analysis of the data was jointly undertaken in repeated sessions with team members to compare, discuss and debate categorization and meaning until a collectively agreed-upon interpretation was reached. For the IDI’s an inductive approach was used to reveal emergent themes that explored participant habitual health seeking behaviors, their needs, suggested best methods for obtaining breast cancer knowledge and solutions to motivate breast cancer screening/early detection services provision in primary care and community settings and active community demand for these services.

## Results

As shown in Table 1, the average age of the women was 47 years with a range of 35–74 years. Most participants had lived in Soweto all their lives, and 62% of them spoke Sesotho as their home language, though participants were multilingual, and all could understand and speak some English. Some 35% had completed high school, 47% had attended but not completed secondary education; 2 had university bachelor’s degrees and 4 had only informal or primary school education. Most women were single (79%), and 91% did not have medical insurance and used the South African public health system which provides diagnostic and treatment services for free, though patients do pay a nominal healthcare facility admission fee.

### Focus group discussion findings

Responses from the FGDs, overlaid on the three domains and sub-elements of the conceptual model are summarised in Tables 2a, b and c.

### Individual domain findings

Most participants were familiar with the word “cancer” and listed various cancers that they had heard of. From a language perspective cancer was understood to be a cyst, sore or worm that does not heal. Causes of cancer perceptions ranged from not knowing the causes to cancer being inherited, caused by infections, viruses, a rash, trauma, cigarette smoking, air pollution, sun exposure, eating unhealthily and being overweight, eating soil, caused by cells growing, caused by taking some medications like aspirin, using soaps, exhausting the breast through breast feeding, an act of God and being bewitched. Signs and symptoms of cancer perceptions ranged from not knowing, cancer perceived as being invisible, perceived through a sixth sense, to signs of continuous bleeding, digestive symptoms, changes in shape, color and hardness of breasts, painful breasts when you squeeze them, feeling painless lumps/stones/knobs/cysts. Understanding of how to prevent cancer ranged from that it cannot be prevented, to vaccination (for children for cervical cancer), detecting it early and with regular self-checking. It was understood that mammograms are used to detect breast cancer and that if it was detected early, it could be cured. Knowledge of treatments ranged from surgery, chemotherapy, radiation treatment which participants described as “*go e chesa*” or “*ukuy’shisa*” which means “to burn it” and some felt traditional healing could cure cancer. It was unanimously perceived to be a fatal disease but there was low perception of susceptibility/or peril as cancer was understood to be a white person’s disease with HIV being a much greater threat to Black people. Participants expressed a great fear of the disease, its treatments and side effects and impact on one’s appearance. The fear of the disease was fueled by perceived high costs of cancer treatment as well as the fear of dying and leaving families, especially children in poverty because of losing their breadwinner to BC.

### Sociocultural Findings

The fear of BC is fuelled by perceptions of internalized, community and health care provider stigma associated with cancer patients. Participants expressed that BC patients exhibit the same physical features as HIV + patients and are thus subject to the same discrimination. BC patients are perceived as being “scary” with a “ghost-like” appearance, and there is fear of catching the disease from those perceived to be infected with it. The perceived self-stigmatisation pertains to being less desirable to their intimate partners and dying and leaving their children destitute. The institutional stigmatisation pertains to chances of losing employment and being badly treated by primary healthcare providers. Culturally, cancer is perceived to be caused by contaminated blood, poisoning and witchcraft. There was ambivalence towards the effectiveness of traditional healers; for people with late-stage disease some participants felt they may help, though their herbal treatments known as “*muthi*”, “*imbiza*/*dipitsa*” may be provided in too strong doses to cause harm and are felt to be rejected by Western hospitals. Similarly, churches were perceived by some as a source of comfort, support, and healing; others felt that some churches reject Western medicines. Participants described the existence of strong social, community and church support structures and stressed the need for personal disease acceptance for individuals to cope with cancer. From a cancer literacy perspective some said they source information from television, radios, clinics, and the internet through google searches but felt cancer information is not emphasized in clinics. Early detection was perceived as the best method to prevent cancer, however some participants expressed that they did not know how to prevent cancer and admitted that they are not pro-active in seeking screening; they wait until they are in severe pain before they access public health services.

### Health System Findings

Although a minority of participants reported positive experiences within their local primary health clinics, participants unanimously reported experiencing clinic nurses as being rude and unwelcoming towards patients. Participants expressed that the poor service from their local clinics was one of the reasons to delay seeking care. Further, local clinics were described as short-staffed, causing long waiting times, patients were not physically examined and that they lack required equipment and often run out of appropriate medication to give to patients. In addition, participants expressed that nurses generally lack cancer knowledge and are not adequately experienced with clinical breast examination.

From the FGDs we found that there is neither a community culture, community facilities, nor community activism for preventative health behavior. Women only access primary health services when they feel extreme pain or ill health. The notion of actively seeking breast cancer screening is seemingly a foreign concept.

We explored these concepts further in the IDIs and individuals’ suggestions for solutions and opportunities to increase provision and demand for breast cancer screening. Our findings are presented as follows:

### In-depth interview findings

From narratives of determinants of individuals’ health seeking behaviors participants confirmed only seeking care when experiencing ill-health, severe pain and/or physical symptoms:

“…*when I feel a discomfort, maybe I’ll get a painkiller or something like that, but if it’s serious, it’s either I go to the clinic or to the doctor.*” (P5)

“… *so, it’s that I am feeling a lot of pain and there’s nothing that I can drink to help me with that pain…*” (P11)

It emerged that financial availability dictated participant health seeking behavior. Most participants expressed that they prefer private healthcare services, but they cannot afford the costs thereof:

“…*doctors are expensive now, so if you don’t have a medical aid, it’s not easy for a person who is unemployed…so, that’s why we mostly prefer to go to the clinic*” (P3), “*I just take myself to the clinic because at the doctor, I don’t have money for the doctor*” (P7)

Participants healthcare provider preferences were based on their perceptions that private healthcare institutions provide better services than public healthcare institutions as they hardly have queues, they operate for longer hours, they always have medication available, and the staff are always friendly and welcoming:

“*You get help fast, you are properly explained to, you’re not chastised, and you don’t wait for a long time to get help…you see, those are the things…. at the private, you’ll find maybe medication, which is not even generic or what, you will find medication which is right…you can see that it will help you* (P1).

“…*but at the doctors, they would give you something different-according to the way they charge you isn’t it…and then if they don’t have that medication, they prescribe the right thing for you so that when you go to the chemist, you get the right medications*” (P11)

In the solution-seeking component of the interviews, we asked what knowledge individuals require on BC. Participants unanimously expressed wanting to know what causes BC, how to prevent it and, what the dangers and/or consequences of being diagnosed with it are. Most participants wished to know how to treat and/or cure it, how treatments affect individuals, including body image and breast-feeding queries. In addition, participants sought clarity on cancer stages:

“…*why does cancer have stages?” (P4), “how many stages does it have?*” (P5)

Participant suggestions on the best ways to receiving knowledge on breast cancer included through television and radio, telephonically via WhatsApp and, face-to-face community engagements:

“…*maybe if they can do like a door-to-door/house-to-house probably in the communities*” (P6), “…*someone who has had it, who knows about it*” (P7), “*Through my phone, so maybe through WhatsApp… have those, you know like your Covid channels whereby you click “say hi” then you get information…*” (P5).

Community campaigns and group discussions which included demonstrations of self-breast-examination (SBE) were greatly emphasized as participants expressed that they did not know or understand how to perform SBE. Knowing how to do so would encourage individuals to routinely perform SBE:

“…*you see as it’s a study like this…if I could go to such a group session to learn about breast cancer*”. (P6)

“*Through campaigns of breast cancer, that’s when I get the information*” (P14)

It was unanimously confirmed that knowledge and awareness of breast cancer does not translate into active self-seeking of breast screening nor of preventative health seeking behavior in general. It was apparent that a preventative health culture does not exist in Soweto communities nor is there any community activism nor community facilities for preventative medicine including early breast cancer detection.

“*Oh okay, screening is something which a person has never done, I haven’t done it and it happened that my daughter had lumps, but they were not cancerous, you see…so but I can say that on my side, I did not go for screening, it didn’t push me enough to go* (P1)

“*You know when we feel pain, that’s when we get up, when we haven’t had pain, we sit…*” (P11)

“…*what I do is that I wait to be seriously sick, that’s when I can go to seek screening but right now, I feel like my breast is fine*”. (P14)

We ended the interviews by asking individuals about suggestions to facilitate cancer screening from individual, community and health care setting perspectives. From an individual’s perspective, most participants suggested that BC and screening should be broadcasted (regularly advertised and talked about)

“…*they could give us pamphlets, maybe while we’re sitting, maybe the nurses should constantly remind us to do screening”. Campaigns. “…even on TV maybe, if there could be an ad that reminds us that women should go for screening, even on the Radio…*” (P13)

Social and community suggestions to activate individuals to seek screening included edutainment whereby it was suggested that there be centers or tents erected where cancer education and demonstrations are given in an engaging and not intimidating way:

“…*and the Safe Hub [a community center in Soweto] as well, there should be a place where activities or sketches that they could do for us which would demonstrate that, about cancer*”. (P4). “…*sometimes when you do those sketches, you remember laughing that “she was saying this”, so that’s something that you will remember, that when they said this…it stays, it gets in the mind…*” (P1).

Participants further called for community meetings about cancer to be held and for children to be taught about cancer in schools:

“…*maybe they could give me pamphlets, I can enter house-to-house, hand them out and tell them that on this date, “we are meeting, there is someone who is coming to teach us about cancer*” (P6). “…*awareness should start early…they should go to schools, from primary to high school to universities…there should be awareness, there should be a vast awareness of this cancer*” (P15).

Participants also advocated for breast and other cancer awareness to be broadly publicized as was Covid-19 on television, radio, and social media e.g., Instagram, Twitter, Facebook as well as a specialized mobile cancer service. In addition, participants also called for regular cancer screening announcements made from the department of health in community settings:

“…*when there’s a roadshow, like maybe in a week, even if it could be once or twice…just a roadshow for people to be reminded that “there’s something called breast cancer” and then we will be able to, as women, screen ourselves while it’s still early*” (P14). “…*for people to be called with a loudspeaker that “come and check, come and check breast cancer*” (P10).

Further, some participants suggested that traditional healers and churches, should work together to encourage breast cancer screening at community gatherings.

“*I see that we trust traditional healers…yes, traditional healers do help somehow because they work hand-in-hand*” (P4)

“…*everywhere where you can find community people gathered, whether it’s at the societies (stokvels), you see, us black people from the township, we are people who are in the community…where people gather, find them right there…another thing is, scream, there’d be a car driving in the township that “women, come, come on a specific day at 2 at a school wherever, at a hall…come, there are things that we are going to teach you”…*(P3)

From the healthcare setting perspective participants strongly suggested that there be specialized cancer centers staffed with providers specializing in cancer and screening.

“*I think it will be better if there could be a hospital for cancer*” (P9). “…*there should be people dedicated to working with cancer only, people who screen for cancer only*” (P15).

Participants also suggested that breast cancer screening be made available at their local clinics and that screening be made mandatory:

“…*they should have it [breast cancer screening] because at the local clinics, they don’t have it*” (P2). “*I think if the doctor could say “it’s a must, go and check for breast cancer*” (P4).

“…*I think they need to check me…they should encourage me; they should teach me why I’m supposed to go for screening*”, (P8)

*” Oh, they [clinics] should have resources to screen…isn’t it…I don’t even know what they screen with*” (P5).

“*I think if the clinic could have, what do you say it is…there are these people who go door to door… get in and say that “we’ve come to check women, if they don’t have breast cancer” …they should just check us, in the houses…yes, they should get people like that*” (P4)

“…*if our clinics had volunteers that would teach people like, locally that “there’s this thing called cancer and then, women, if you want to screen for your cancer, you go to a place like this, at a time like this, daily basis”, that when I think that it will be better, yes*” (P14)

Last but certainly not least, participants suggested that the staff at the clinics be increased and that there should be volunteers who teach people about breast and other cancer and, that there be a change in staff attitude including a faster working pace:

“…*they should change their attitudes*”, (P10). “*I think they should do things faster*” (P3), “*they could be nicer*” (P3). “…*they should increase the nurses*” (P9). “…*if our clinics had volunteers that would teach people*” (P14).

## Discussion

In summary, participants had poor health literacy of breast and other cancers. Most participants perceived cancer as fatal and expressed great fear of the disease although there were perceptions of low susceptibility and misperceptions regarding manifestations of the disease. From a sociocultural perspective, the fear of BC was fuelled by perceptions of internalised, community and health care provider stigma. Further, there was ambivalence towards the effectiveness of traditional medicine and/or healers in treating cancer, although the church was perceived as a powerful source of emotional and spiritual support. The primary health care system was generally perceived to be unwelcoming and ill-equipped to deal with breast and other cancer symptoms. Overall, the notion of preventative health seeking behaviour was absent and fuelled by competing socioeconomic priorities. Consequently, women reported only seeking health care when experiencing extreme pain or ill-health.

These findings aligned well and provides evidence towards the conceptual model used. Surprisingly, though our participants were familiar with some common cancer risk factors, none of them mentioned older age as a major risk factor for breast and other cancers. Furthermore, most participants were familiar with and practiced routine pap smears at their local clinics but very few participants knew of and/or had ever done a mammography. In addition, there were no reported activities and mechanisms within the community to encourage and support preventative health seeking behaviour such as breast and cervical cancer screening, despite the mandated cervical cancer screening program ongoing in South Africa.

Evidence from this study and others suggest that there is a prevailing lack of knowledge of breast and other cancers and developing the disease among Black women[8, 9, 13, 14, 17, 18, 24, 35, 36]. In HICs, poor knowledge of breast cancer is also prevalent among Black and other socioeconomically disadvantaged minority populations[11, 37–39] where comparatively lower educational and health literacy levels and higher levels of fatalism persist[8, 37]. Such findings were confirmed amongst our participants who were mostly unemployed but had completed secondary schooling but relatively low levels of tertiary education.

In African cultures, cancer is conceptualized in a different way compared to the way in which it is explained and understood within Western countries. In our study, cancer was reportedly known as *mdlavuza, isifo se phepha, kankere* or *siso se sa foleng* in isiXhosa, isiZulu, siSwati and Sesotho and Setswana respectively. Participants who were natives of Lesotho (a neighboring country of South Africa) knew of cancer as *mofese* and breast cancer as *mofese wa letswele*. Some participants explained cancer to be caused by dirty or contaminated blood with the treatment thereof being *imbiza/di pitsa* which is traditional herbs or mixtures mostly concocted by traditional healers. Some participants explained that witchcraft can also be used to cause cancer. This echoes what other African studies have reported on the causes of cancer. For example, cancer has been reported to be caused by a higher power such as God or supernatural forces like witchcraft and/or ancestral spirits. In these settings, some women were reported to seek alternative treatments, including from traditional healers who provide traditional medicine and are believed to know how to tackle supernatural forces[25, 40, 41]. Similar sentiments were echoed among studies conducted with Korean American women in the USA, Arab-Palestinian women, and Malaysian women[12, 17, 42]. In contrast, our participants reported some ambivalence around their beliefs of the effectiveness of traditional healers in the management of breast cancer.

Almuhtaseb and Alby[12] found among Arab-Palestinian women that fatalistic beliefs about breast cancer caused resistance to seek care. It was believed that it is useless to consult with health care providers because once cancer is diagnosed, death is inevitable. Religious notions of breast and other cancers in LMICs have been powerfully promulgated by religious and local opinion leaders[43]. While culture have been found to be highly influential in many women’s health seeking behavior, in South Africa, where cancer treatments are provided at no cost to patients treated in the Public Health sector, this notion is changing. Our own findings along with another from the KwaZulu-Natal province of South Africa, Zwane[36] suggest that many South African women no longer believe that traditional healers are effective against breast and other cancers. Further, our participants generally viewed the church as a source of comfort and emotional support rather than providing actual healing. Similar sentiments regarding religion and spirituality were echoed in Daher’s study[15] which reported that many individuals in Middle Eastern countries rely on their faith and spirituality as a coping mechanism when confronted with an illness.

Social stigma powerfully perpetuates myths and misconceptions about cancer, which possibly causes delays in patients’ health seeking behavior[10, 15, 44]. As Zwane[36] argues, uncommon diseases such as breast cancer are not easily recognized and/or acknowledged in many Black communities. Consequently, breast cancer patients are subjected to social stigma in the form of excess pity and isolation from those who perceive the disease to be infectious and confused with HIV/AIDS. The impact of stigma consequently fuels the pervading fear of cancer and a cancer diagnosis[19] causing resistance to seeking care in the face of visible cancer symptoms, or denial of symptoms by blaming other ‘unseen causes’[43]. From an economic perspective, “out of pocket” costs to access care also contribute to the delay in detection and diagnosis[44].

Health care providers, especially those in the primary health care setting play an important role in women’s perceptions of breast cancer and attitudes to breast cancer[45–50]. Our participants expressed perceptions of provider inadequate knowledge of and technical skills to recognize and manage breast and other cancers and their too often unwelcoming attitudes towards patients. These perceptions were confirmed by providers from 8 primary care clinics in and around Soweto[51], it was also reported that their clinics lacked the required screening facilities to perform clinical breast examinations. Inadequate provider knowledge and infrastructure have also been documented in several other studies from both LMICs and HICs[45–48, 52], with insufficient advocacy for political and social action in the field of cancer control[53]. Thus, structural vulnerability affecting individuals, households and communities are powerful social factors that impact access, diagnosis, treatment, and outcomes of cancer [54]. There is also limited advocacy for political and social action in the field of cancer control[53].

Solutions for increasing screening uptake suggested by our participants included: (i) sustained community interventions by the primary health care providers working collaboratively with existing community resources, and (ii) continual encouragement to undergo screening. From an individualistic and community perspective, suggestions regarding cancer awareness campaigns included health literacy campaigns whereby women are taught how to perform self-breast examinations (SBE) through physical demonstrations. Participants expressed that being taught through physical demonstration would clarify what exactly it is that they need to do and look out for when performing SBE. Educational group gatherings at community centres/halls and/or sportsgrounds were also suggested as another way to teach community members about breast and other cancers. Participants further suggested that there be house-to-house visits by health professionals to educate families about cancer. Participants suggested that there be constant announcements promoting breast and other cancer awareness as has been previously done in the community with other illnesses such as HIV/AIDS, TB, and the most recent, Covid-19. From the health system perspective, participants suggested that there be specialized cancer centres in the community as this would enable people to walk in and query about breast and/or other cancers without having to navigate the local clinics. Participants further justified this suggestion by acknowledging that local clinics and staff are heavily burdened by competing priorities, therefore, specialised cancer centres in the community would ease the load off clinics while also encouraging people to go for screening and/or any other cancer related queries thereby alleviating long queues and the possibility of being turned away by clinic staff for presenting with what might be perceived as “not urgent” issues. In addition, participants suggested that nurses teach and encourage women to routinely practice SBE and that clinical breast examination be made mandatory for all women by clinic staff, especially doctors.

Our study limitation is that the results may not be generalizable to other urban and rural areas of South Africa. They need to be tested against experiences and perceptions of other women, communities, and policy stakeholders. Overall, a combination of both FGDs and in-depth interviews provided more robust and consistent findings.

### Implications

Our findings have implications for future interventions to better manage breast and other common adult cancers in SA and other resource constrained settings. The prevailing cloud of misunderstanding about breast and other cancers among women from socioeconomically disadvantaged communities fosters confusion, socio-culturally influenced misperceptions, fear, and emotive stigma about the disease. This in turn potentially negatively impacts cancer screening and other preventative health seeking behavior among women. Unlike for HIV/AIDS where great strides in management of the disease have been attained, there is an absence of clear, consistent, and sustained messaging about breast and other common cancers (and for that matter other common noncommunicable diseases) for South African communities. As suggested by our own participants, sustained community engagement is needed through existing community resources to educate the population about breast and other cancers and to encourage them to actively seek screening for early symptom detection. This requires primary care clinics to harness and work hand in hand with churches and religious leaders, traditional healers, community leaders and community facilities. But to be sustainable, the necessary political will and support from policy makers is required to address the inadequate cancer knowledge of primary care providers and to support and adequately resource their much-needed community outreach activities.

## Conclusion

In conclusion, multiple factors influence how women may perceive disease and act upon to seek health care. Given that the cancer health burden will likely increase in South Africa it is critical that we develop sustained community interventions, utilising clinic and community resources working together to provide clear and consistent messages about breast and other common cancers and to encourage communities to access screening and other preventative health services.

## Figures and Tables

**Table 1:** Characteristics of focus group discussion participants

Variables	N = 34
Age (years), mean (range)	47.0 (35–74)
**Years living in Soweto, N (%)**	
Their whole lives	24 (71)
20 or more years (includes whole lives)	30 (88
5 and 13 years	3 (9)
**Home language, N (%)**	
Sesotho	21 (62)
Setswana	6 (17)
isiZulu	5 (15)
Sepedi	1 (3)
Siswati	1 (3)
**Level of education, N (%)**	
University graduate	2 (6)
Completed high school (matric)	12 (35)
Some high school	16 (47)
Primary school or informal education	4 (12)
**Marital status, N (%)**	
Married or in a relationship	7 (21)
Single or divorced	27 (79)
**Has Medical Insurance, N (%)**	
No	31 (91)
Yes	3 (9)
**Has accessed public health services, N (%)**	
Yes	31 (91)
No	3 (9)

**Table 2a.** Individual Findings

**INDIVIDUAL DOMAIN**	
**Knowledge and awareness of cancer and breast cancer**	
• Types of cancers	o Brain tumour, womb cancer, breast cancer, throat cancer, blood cancer-FGD 1 o Breast cancer, blood cancer, cervix cancer, stomach cancer, “we name them, but we don’t know how to explain them.”-FGD 2 o Prostate cancer, Breast cancer, Leukaemia-FGD 3 o “It’s cervical, it’s throat, it’s one on the gums, it’s brain tumour, right?”, Leukaemia, Bones cancer, lung cancer,-FGD 4 o “Of the breast”, “Of the blood”, “Of the cervix”, “Of the throat”, “Anal…or what is it…the one of the backside, is it anal or, no, of the rectum or what is it…it goes along with the intestines on the backside whereby you find it hard to poop, there’s one for skin, there’s one for, like thyroid, there’s one for brain”, “On the legs”, “Mouth cancer”-FGD 5
• Perceived as a deadly disease.	o “Cancer is a harmful disease from our bodies”-FGD 1. o “It’s a silent killer”-FGD 2. o “Cancer is uncurable, that’s what I know”-FGD 1,4
• Cancer causes	o “I don’t really know what causes it, but it is also painful [physical and emotional] because it is just not visible, it is not visible”, “^…^as black women, we have no idea what causes cancer.”-FGD 1 o “…it’s being said that if there’s a person within the family who has cancer, it spreads, so, it is something that people in that family always get.”-FGD 1 o “The one of the blood…the one of the blood is like, sometimes, it is hereditary…yes, let me say, maybe it starts by, when your blood, yes, the blood one tends to, sometimes, you are born with it…it could be maybe hereditary or just by nature, you’re born with it when you’re a child”-FGD 5 (FGD 2) o “Through infections, I’ve heard so many cases of cervix cancers and most of them they say that they develop from infections.”-FGD 2 o “Me the way I know it, in the body, there are things that are called cells isn’t it, that combine the skin to do whatever, so, when there’s a problem with those cells, they tend to grow in a surprising way…like, let me say like, the skin, the skin grows as you grow right, so you find that at that time, those cells, they grow more than enough…so, that is when they cause problems in the body, then, they eat whatever there where they shouldn’t be.”-FGD 4,6 o “Me I think it’s a disease, it’s one of the diseases that are caused by God…”-FGD 4 o “You were bewitched, got cursed, *muthi was* used, so, meaning that it is spiritual…they think that it is spiritual because of, you’ve been bewitched”-FGD 2,3,5,6 o “The one of lung cancer I would say that it is caused by cigarette… maybe when you have a problem of the lungs…yes, a cigarette because it has nicotine, it is what can cause you problems.”-FGD 5 o “It’s like, not eating healthy food…and then also by not, it’s like, having a big body, being fat…”-FGD 2, 3, 4, 6 o “If you get an accident and find that your blood ran to your brain, just a blood clot, if it gets inside your brain, you get brain cancer” FGD 5 o “Like I was saying that the likes of flu, and then you find that maybe it causes a rash, when it doesn’t heal, maybe it the one that causes that thing”-FGD 6 o “I tend to hear it being said that like soap, us females…like, when we bath our private parts, we use water that has soap and then that soap can affect us and cause us womb cancers.”-FGD 3 o “Probably, the breast has had enough, plus isn’t it us women, we breastfeed…so, the veins have had enough with taking out that milk”-FGD 6 o “Others are caused by Grandpa’s [Pain medication] …when you drink Grandpa a lot.”-FGD 5 o “Cancer is just a virus that emerges out of nowhere, that’s what I think.”-FGD 2 o “I say that it is caused by bacteria, maybe air pollution what is it… where it is messy, you find that you’re always working there and then you get infections, sometimes, you find that you work hard there, you end up getting after infections.” FGD 2 o “The likes of soil” (edible soil)-FGD 2 o “…then when that sun burns you, you find that it creates other problems for you, or it is cancer”, “I tend to hear that it’s caused by the sun”-FGD 3
• Signs and symptoms of cancer and BC.	o “Especially the womb one [cervical cancer], you see by bleeding endlessly, on your vagina”-FGD 3 o “The one of the stomachs, maybe you find that you’re unable to defecate, there will be blood coming out or maybe your intestines, it’ll be hard for you to digest, for food to be digested”-FGD 5 o “It’s the breasts that are uneven, one becomes big, one becomes small, and when you touch it, you find it has hardened”, “It is with the lump, it’s like, when you touch the breast, there is something inside, it is like a stone, it is hard, and while you press, it pains… you find that even your nipple changes colour or the breast changes colour and it’s painful…so, those are some of the things that…you see.”-FGD 6 o “Under the armpits, especially for the likes of breast cancer, it can be caused by lumps”-FGD 1, 3, 6 o “Your nipple changes, there’s also those discharges that I said come out and it becomes painful…when you feel any discomfort, you will feel that, and there is this thing that they call 6th sense, you listen to your body, your body will tell you when there is something wrong…then go and check it out”-FGD 6 o “Sometimes you’ll see tumours emerging, and lumps on the body and then they cause you a serious disease”-FGD 3…. “It’s mostly said that when you feel yourself, you feel a lump.”-FGD 2 o “When they explain it at times, they say that you don’t feel pain with cancer, but there are symptoms that happen with you. You don’t feel pain but you can feel that there is something like a knob or something but there is no pain, you only feel it when you touch yourself but it’s not painful”-FGD 1,2
• Cancer prevention	o “So far, they are not visible…they are not visible as in when I get flu, I know that I will get a runny nose and all that…so cancer, I think maybe sometimes if I say I’m sick and I go to the hospital and they do a check-up on me, it is how I will know that I have cancer.”-FGD 1 o “Me I know it as a cyst, like it starts as a sore right, and then it gets pus right, and then when it bursts, that’s what causes that cancer.”-FGD 4
• Early detection of cancer	o “I think that when you’ve found it early, you are able to confront it, I think when you find it early, you’re able to get a cure early.”-FGD 3, (FGD 1), (FGD 4) o “Even now, children get injected for cervix cancer [HPV vaccination].”-FGD 2 o “I will stay checking myself, even sexually, you try by all means to be protected, using protection and stuff like that.” FGD 2, (FGD 4) o “I say that it can’t be prevented, even this one for the children, they are not sure…okay, no, it can’t be prevented, if you’re supposed to be getting it by that time, even if you can get an injection however many times, drink the pills thereof, you will get it”-FGD 6
• Cancer cure, treatment, and management.	o Yes, at the mammogram…let’s say you cry about pains on the breasts, anything that goes with the breast, pain, even when it doesn’t have lumps, you can say “doctor, I’m feeling pain on the breast"…so, they send you for a mammogram, mammogram is the one that will say what is happening…how far is your cancer, is there or is it not there.”-FGD 3 o “If they detect it early, it’s possible that they can remove the lump. And then, even when you go for treatments, you might find that it can, through treatment, it can help you”-FGD 2 o “It can be cured if they find it at an early stage”-FGD 4 o “Those radiations and chemo’s”, “We know of that chemo, that when a person has cancer, it goes by stages…I don’t know how chemo works but we always hear of chemo, that it is the cure for cancer”.-FGD 2 o “…they will see what stage it is on…then there are stages that when they are…or early, it’s like they do radiations, what else is it, the one that burns?”-FGD 3 o “Chemotherapy, “I was saying that they burn those cells because those cells are ones that would’ve grown out of control, they would’ve caused the cancer to be there”-FGD 4 o “…they cut off the affected part.”-FGD 2 (FGD 3), (FGD 4) o “She has healed, but she was helped by some lady from Rockville [an area in Soweto] …they say that she has a traditional mixture for it…they say you buy it, you buy 3 of them for R150-00, you cook them and drink them.” FGD 4…… “When you go to traditional healers, and they give you medicines or going to the doctors.”-FGD 4
• Language/Cultural context in understanding cancer-not an enabler per se	o “*Siso se sa foleng*”-a cyst that doesn’t heal. Siso can also mean a sore, ulcers or abscess. o “*While growing up, they used to say a cyst that doesn’t heal and eats you*”-FGD 3, 4, 5, 6 o “Cancer is known as *kankere* and/or *mofese in* Sesotho, and it is known as Mdlavuza, isifo semhlaza, and/or isifo se phepha in isiZulu & isiXhosa. It is treated with traditional mixtures/herbs called *Imbiza* (plural), or *mbiza* (singular) in isiZulu and isiXhosa, and is called *Dipitsa* (plural), or *pitsa* in Sesotho. Mbiza is more common as it is also used by non-Zulu & Xhosa speaking people
• Cancer as a worm	o “Cancer is like…what can I say, it’s a small worm, it eats… It’s like, it’s this worm…*yoh*, it’s a small-ish worm that is small, but you won’t be able to see it…yes, it moves around on your body, then, wherever it is that it stops, it eats”-FGD 5, 6
• Language as a barrier in communication	o “Me most of the times, if I see that they are saying words that I can’t understand in English, me I ask them to explain to me what they mean”-FGD 4
• Language use in understanding radiation therapy	o “We mean to go and get burnt, to go so that they can burn her.”-FGD 4 o …it’s been said that they burn the breasts and check if they are coming alright.”-FGD 1 o …they prevent it by burning it…”-FGD 5
**Perceived susceptibility (risk of getting the disease)**	
• Family history	o “…this thing is tricky because you might find that okay, grandma had it but now, moving forward, nobody has it; so, you’d never know that triggers that or is it the chromosomes that they talk about or whatever that makes you have that breast cancer or any other cancer”-FGD 2 o “Me what I understand is that sometimes people who have cancer, you find out that in the family, it comes from the family…some of the family members had had it; so, it’s likely that even when a child comes-sometimes-will take some of the, you know, what do they call them? Those heredities in there.”, “It’s the abnormality cells”-FGD 3, 5
• White People’s disease	o That is why we black people say…cancer, it’s a disease for white people”-FGD 1, 4 o “…my understanding was that it’s a white people’s disease, not a black people’s disease. So, it’s only recently that we know of cancer, even so, we don’t really know…”-FGD 2, 6
• HIV perceived as the major disease threat rather than cancer.	o “We don’t understand it and even now, we don’t believe it, right now, we believe that HIV is the one and we even know a way to cure it.”-FGD 1; “…they always think is that everyone has HIV…”-FGD 4 o “I don’t think there’s too much exposure towards cancer because right now, people are focusing on HIV” FGD 2
**Fears of cancer treatments and effects**	
• Fear surgical treatment.	o “…the people who have been cut, I know that they die…I’ve never heard of one who has survived after having their second breast cut…so, I don’t know if they can, that’s the thing that I don’t understand.” FGD 1,4 o “It is scary because sometimes, you find that-there’s a woman I know, she had cancer of the breast, they were going to take it out, so they cut the wrong vein, she died with her hand big and rotten” FGD 2
• Fear of treatment side effects	o “Some it doesn’t make you weak, but some people when they leave there *iyho,* they are miserable.”-(FGD 2) o “They say that they burn it because a lot of people who I know, they used to say that when they are going to the hospital, they are going to burn it, but it seems like it doesn’t get better, it keeps getting worse”-FGD 5 o “…they don’t come back being better when they return from chemo, they come back being weak …she comes back having changed, weak, she’s tired and whatnot…not wanting anything…she would just want to sleep, she struggles when she goes to the toilet…she cries…when she comes back from chemo, *haii*”-FGD 5
• Fear of treatment impact on appearance	o “Changing colour and hair that shreds *yoh"*-FGD 2, 3 o “You know as I was saying that I have an ex-colleague of mine who had cancer, her hands would turn black…I would ask myself that, how does it happen for the hands to turn black.”-FGD 5
**Perceived urgency and peril of cancer**	
• Fear of death caused by the disease.	o You have a fear that “I’m going to die”-FGD 2, 3, 4 o “Cancer makes a person to always be scared, when you have cancer, you’re already thinking that your days have shrunk”-FGD 1 o “I am afraid of it to speak the truth”, “I am also scared of it”-FGD 2,6 o “…the people who have been cut; I know that they die…I’ve never heard of one who has survived after having their second breast cut.”-FGD 4
• Fear of disease expenses	o “Maybe it is expensive…maybe the treatment is expensive”-FGD 4 o “I’m not able to get the things that I want and the food as I mentioned earlier that “if I had money, I would be able to buy those pills when they say they can help.”-FGD 2 o “Any and everything is money”-FGD 6
**Stress**	
• As breadwinners the worry about dying and leaving the family destitute	o “When you start worrying, worry affects you. You say “*yho,* I’m going to die, who will my children be left with” this and that…at work, you no longer go properly, you be absent, you say “they will fire me, I’m no longer going to work, they will look for another person”-FGD 2 o “That person can have depression That, how to face her family…and the world, have you seen…that how she will do”, “*Yhoo,* it’s undergoing all the treatments.like, she can clearly see that she will die soon soon.”-FGD 4

**Table 2b.** Sociocultural Findings

**SOCIOCULTURAL DOMAIN**	
**Stigma**	
• Societal stigma-confusing cancer symptoms with HIV symptoms	o *Iyho,* in most of the cases, they never think of cancer…cancer is the last disease that they think of. When you lose weight, lose hair, having symptoms that are associated with HIV but having cancer, they will never think of cancer.”-FGD 4, 6
• Social discrimination	o “They are scary” [people who have cancer]-FGD 3 o “…we don’t have information, people don’t talk when they have it and you can’t see that they have it…you’ll never know what is going on, they live a normal life”-FGD 1; Some of them hide themselves, they are afraid, maybe rejection…they are scared of being rejected.” FGD 4 o “Let’s say that a person’s mentality, when someone says that they have breast cancer, to be honest, the first thing that comes to mind is death” … “That person is leaving…she’s kicking the bucket…yes”-FGD 4
• Fear or catching the disease of partners/spouses.	o “…the man of the house will no longer want to touch me… he is scared that he will get cancer because now I am dying”-FGD 1 o “…will she not infect me as I am living with her… as she calls me to go help her, I come running to go help her, I don’t have gloves I don’t have anything, maybe this thing of hers will also get me”-FGD 3 o “They will change towards me, won’t treat me like they used to at first.”, “They will judge me.”-FGD 2
• Self-stigmatization	o Even marriage, your person…yes, indeed it’s obvious that a woman’s body is her confidence, you see…so to your person, let’s say you’re going there in the bedroom, bedroom things there, you’re scared to undress, your 1 breast is not there, one is there.”-…. “Less woman” FGD 3, 4, 6 o “…you feel guilty that “I will wake up and die, I’m going to die.”, “When they tell me that it is possible for it to be treated, my conscience will be down, it will be better, and then I will still live, and I will also do my best to comfort myself that “I’m still going to live for my children”-FGD 2 o “It becomes mine; it is my secret that will eat me up because we believe that cancer kills.”-FGD 1
• Institutional stigma: Employer and Health Care providers	o “…. they will fire me, I’m no longer going to work, they will look for another person” FGD 2 o “Why do you want us to check you, we gave you pills, you’ll come back another day” [upon requesting to be screened]-FGD 2… Yes, they chase you away and say, “go home”, “You’ll come back the day you know what you have” [after informing them that you self-detected a health symptom] FGD 2 o “When they haven’t closed, they are still inside but you will think for yourself…they say “*iyho,* you come at this time, who do you think will attend you?”-FGD 4
**Culture, religious beliefs, and fatalism**	
• Cancer is caused by dirty blood.	o “Sometimes they say that it is dirty blood, that cyst is caused by blood that is dirty-means that your blood is now dirty, it has created an internal cyst and then you rot inside”-FGD 1, 3
• Food poisoning	o “….. or it can come out as cancer at the hospital, but they might not know how to treat it” … “Meaning it can come from *sejesong* (sejesong means food poisoning)-FGD 2
• Witchcraft	o You were bewitched, got cursed, *muthi was* used, so, meaning that it is spiritual…they think that it is spiritual because of, you’ve been bewitched.”-FGD 3,4
• Church as source of healing but also resistance to Western medicine	o “Isn’t it as long as you have faith that “when I pray, I will heal” … you will pray and heal”, “But some churches don’t want that isn’t it… when you go to them, they don’t want these doctors’ things and what, they only want to pray for you”-FGD 3 o “When you believe that you will be healed, when you believe that “this sick should leave”, it will leave” FGD 2 o “And when I get inside the church, I meet the pastor, the pastor will pour water for me and put hands on me”-(FGD 2)
• Role of traditional healers for good and for harm	o Yes, some doctors may never see a thing called *sejeso* (poisoning) you know, so they will tell you to…”, “They are important because, even these people, like doctors, when they are stranded, they tell you to go to a traditional healer.” FGD 4 o “And the painful thing, they treat something they don’t know…isn’t it cancer has stages, has this and that…maybe you’re on the last stage, they give you strong things that will make you leave sooner”-FGD 2. “I would say that traditional healers, they heal something that they don’t know, that they only imagine how it is.”-FGD 3 o “Sometimes, challenge number 1, they tend to overdose, when they say they give you *mbiza,* you find that they give you this much, you drink a lot, it doesn’t have grams, doesn’t have what…you just drink, 1 litre, you end up overdosing, you burden your body with the overdose of the *mbiza* that you were given”-FGD 3 o “isn’t it sometimes you find that you’re admitted at the hospital, and then, you find that they bring you stuff from home, the hospital says “no, these things are not allowed in.”-FGD 4, 6
**Health Literacy**	
• Contrasting opinions on cancer information available to communities	o “Like television, even radio stations, they tend to call an expert to explain, even Google, you can get information”-FGD 2 o Sometimes when we’ve gone to the clinic, there are awareness’ [talks]”-FGD 3 o “Look right now we have Covid, they talk about it…HIV, about Aids, but cancer, there’s less awareness with it…you know, because yes, we hear that there’s a lot of them, but we are not even having ideas about the symptoms thereof, how it should be treated…like, things like that…like, we are clueless”-FGD 5
**Perceived benefit of preventive action**	
• Early detection/diagnosis will lead to a longer survival rate	o “If they detect it early, it’s possible that they can remove the lump. And then, even when you go for treatments, you might find that, it can, through treatment-it can help you”-FGD 1 o “It hasn’t spread, it can be cured”-FGD 3
**Perceived barriers to preventive action**	
• How it can be prevented is unknown; Preventive health seeking is not practised	o “…we don’t know how it can be prevented; I don’t have information on how it can be prevented…”-FGD 1, 6 o “I don’t know, especially with breast cancer”-FGD 2 o “We often relax”, “We don’t go to the doctor”-FGD 4
• Only go to the clinic when they feel extreme pain	o “I don’t go anywhere if there’s no pain…I’ll keep saying “lump, I’ll see you some other time”, but when it starts being painful, that’s when I’ll jump and say “this thing is not making me sleep”-FGD 1, 2
**Economics**	
• Being financially stable will enable a person to afford better healthcare services (private healthcare system), food and transport.	"Because remember that you need to eat specific food…you can’t eat foods that has fat, maybe it’s a diet that is strict, you need to eat certain foods, obviously if you don’t have money, it won’t be possible… I’m not able to get the things that I want and the food as mentioned earlier that “if I had money, I would be able to buy those pills when they say they can help”-FGD 2 o “Mam V maybe was able to get help because she went to like, private doctors, I just don’t know how if and how far public would help her because of our hospitals don’t ever have equipment”-FGD 5 o “…everything is expensive and then, these people, like, when you go to the privates, and cancer mostly is treated in the western way, you understand…and it’s as if the western way is what deals with it best, though we still drink our mixtures, so, people who have medical aids, obvious, you will go to landmet (Lancet laboratories) and go wherever…”-FGD 6
• Money won’t save a person from dying from cancer.	o “Having money, no, it won’t help you”-FGD 3, 6 o “When it has eaten you, you go, you will leave that money behind.” FGD 3
**Social support structures**	
• Individual (Self-acceptance to cope)	o “The first thing is to accept yourself, that is the first thing before you go out to the society, you have to accept and talk to yourself that “I have a sickness like this, I must accept it”-FGD 2
• Family	o “…your friends and family ought to be with you through that thing that you’re facing because you might find that some families or some friends-they be with you just now, not throughout the whole process”, “you go to your family, family also counsels you, they guide you, things like that then, your friends also sit with you and comfort you while on your side…that’s how I think you can be okay”-FGD 2
• Community (e.g., Church)	o “…with religion, obvious, they pray for you, you pray, you also have to have a believe…so, at churches, they pray for you according to you, like they pay for you, they will say “come, let’s pray, let’s pray for the sickness that you have to go down” at that time you also have faith that you will heal through prayers”-FGD 2 o “Prayer, you only use it when you’re going to do an operation that “Father God, please help the doctor so things can go well” …it’s how it will work”-FGD 3, 4, 6

**Table 2c.** Health System Findings

**HEALTH SYSTEM**	
**Perceptions and feelings about the health system**	
• Feel uncomfortable due to ill-treatment by clinic staff.	o “*Ei,* nurses are rude, especially the ones at public clinics…they are rude!”-FGD 6 o “I don’t feel comfortable, honestly because you would go to the clinic, and you tell them that “I suspect that I’ve got this” and then they say, “where do you know that from?”-FGD 1–6 o “If I had a choice, I wouldn’t go”-FGD 5 o “The nurses themselves, they make you feel uncomfortable when they ask you what you came for”-FGD 2
• Feel intimidated to ask questions.	o “Nurses have an attitude, and when they see that you are a questionnaire (ask a lot of questions), they don’t even want to see you in front of them”-FGD 1
**Access and availability of staff and facilities**	
• No doctors at the local clinics, only at Community Health Clinics, secondary and tertiary hospitals	o “Our clinic… hardly has doctors”.-FGD 1. o Doctors are not there, even at Moroka clinic [primary health clinic] …they are only at Chiawelo only [Community Health Clinic].”-FGD 1-6
• Clinics have staff shortages; nurses don’t spend enough time with patients.	o I think that those people are short-staffed, they burn out…like, they are human beings, when you work with a lot of people in a day, you understand that there should be a limit that at least in one day “I work with this many people”-FGD 2, 3 o “*Haii,* sometimes they always complaining that they are short-staffed, one is absent, there’s a staff shortage, doctors are not there, they went to the hospitals.”-FGD 1–6
• Clinics don’t have enough facilities and equipment and medication.	o “…they will just give you Panado, Ibuprofen or Allergex [pain, anti-inflammatory and allergy medications only available]-FGD 1 o “…we queue for nothing, no medication”, “They will tell you to buy lemon, they don’t have what, they don’t have what.”-FGD 4 o “We already know that when you go to the clinic, they give you Maximax, they also give you Ibuprofen…and Allergex”-[pain and anti-inflammatory medication] FGD 1–6
**Personal experience within the health system**	
• Spend a lot of time queueing and waiting for service.	o “…you wait for them when you go in there…it’s like, they work at their own pace those people…”-FGD 4 o “…you sit there at the stretch for a very long time, the Sisters will tell you “hey, we’re short of doctors, don’t annoy us you”-FGD 2 o When they haven’t closed, they are still inside but you will think for yourself…they say “iyho, you come at this time, who do you think will attend you?”, “They are just angry, they are annoyed that they can see the time, what time it is, “why are you only coming at this time?”-FGD 4.
• Being chastised and badly treated by nurses and clerical staff.	o “Sometimes you find someone who is fine, sometimes you find someone who just gets irritated and throws their hand at you.”-FGD 3 o “Right now, do you see how old I am? When I go to prevent, they say “these grannies don’t want to finish/be done” … so should I now make babies because I am sexually active? And if you chastise the person, they will say that you are disrespectful.”-FGD 1 o “*Aiih,* they illtreat us these people, it’s like they are not human beings as black people, no…you know, even the doctor, the doctor is lenient with you, but nurses! Clerks! No, they don’t know how to treat people well, the doctor is better” “-FGD 1–6
• Not being thoroughly examined-no physical contact since Covid-19 precautionary measures were implemented.	o “They don’t check you.”-FGD 2 o “Covid helped them [helped the nurses], it made things worse.”-FGD 1 o “Unless you insist that “check me” and yet still, they tell you nonsense…remember before, we used to get Sr that when you get there, she would check your chest with a stethoscope and say, “this is where something is wrong”. Now, they don’t care to a point that you get there “okay, what do you have?”, “here are pills, get out and leave” and then that’s it”-FGD 2
• Some participants reported having had a pleasant experience.	o “To be honest, clinics differ, they differ because me, at the one in Moroka [community clinic], when I used to take my child, it was, what…it was fine…”-FGD 3 o “But sometimes, you do find somebody who is fine”, “…some Sr’s are fine, and you understand, they even sit down with you and talk as if she’s not a Sr”-FGD 2
**Cancer education and awareness of staff**	
• Nurses don’t know much about cancer.	o “…they shout because we will ask them questions that they won’t be able to answer, they did not go to school”-FGD 1 o “I’ll tell you what one Sister once did to me…and the way she’s so friendly, you all know her for sure…she’s dark, she’s short…so, I had a pain here, she was from eating, it’s lunch, so she asks what I have, I say “No, it’s painful here”, she then said to me “*hai hai hai,* don’t tell me that thing, I just finished eating!”-FGD 3 o “…some of them don’t know, the nurses…but when they see that you’re nearing death, they send you to Bara”-FGD 6
• Clinical breast examination is not offered at local clinics.	o “At the clinic, if you say, “my breasts are painful”, that’s when they check you…they don’t just check”-FGD 3 o “What is a mammography now?”-FGD 6 o We don’t know it”, “We just hear of it when people talk.”-FGD 4

## Data Availability

The datasets generated and/or analysed during the current study are not publicly available due to privacy ethical agreements with participants but are available from the corresponding author on reasonable request.
